# Healthcare providers balancing norms and practice: challenges and opportunities in providing contraceptive counselling to young people in Uganda – a qualitative study

**DOI:** 10.3402/gha.v9.30283

**Published:** 2016-05-11

**Authors:** Mandira Paul, Sara B. Näsström, Marie Klingberg-Allvin, Charles Kiggundu, Elin C. Larsson

**Affiliations:** 1 Department of Women's and Children's Health/IMCH, Uppsala University, Uppsala, Sweden; 2 Department of Women's and Children's Health, Karolinska Institutet, Stockholm, Sweden; 3 School of Education, Health and Social Studies, Dalarna University, Falun, Sweden; 4 Department of Obstetrics and Gynaecology, Mulago Hospital, Kampala, Uganda

**Keywords:** contraceptive counselling, young people, health service provision, induced abortion

## Abstract

**Background:**

Pregnancies among young women force girls to compromise education, resulting in low educational attainment with subsequent poverty and vulnerability. A pronounced focus is needed on contraceptive use, pregnancy, and unsafe abortion among young women.

**Objective:**

This study aims to explore healthcare providers’ (HCPs) perceptions and practices regarding contraceptive counselling to young people.

**Design:**

We conducted 27 in-depth interviews with doctors and midwives working in seven health facilities in central Uganda. Interviews were open-ended and allowed the participant to speak freely on certain topics. We used a topic guide to cover areas topics of interest focusing on post-abortion care (PAC) but also covering contraceptive counselling. Transcripts were transcribed verbatim and data were analysed using thematic analysis.

**Results:**

The main theme, HCPs' ambivalence to providing contraceptive counselling to sexually active young people is based on two sub-themes describing the challenges of contraceptive counselling: A) HCPs echo the societal norms regarding sexual practice among young people, while at the same time our findings B) highlights the opportunities resulting from providers pragmatic approach to contraceptive counselling to young women. Providers expressed a self-identified lack of skill, limited resources, and inadequate support from the health system to successfully provide appropriate services to young people. They felt frustrated with the consultations, especially when meeting young women seeking PAC.

**Conclusions:**

Despite existing policies for young people's sexual and reproductive health in Uganda, HCPs are not sufficiently equipped to provide adequate contraceptive counselling to young people. Instead, HCPs are left in between the negative influence of social norms and their pragmatic approach to address the needs of young people, especially those seeking PAC. We argue that a clear policy supported by a clear strategy with practical guidelines should be implemented alongside in-service training including value clarification and attitude transformation to equip providers to be able to better cater to young people seeking sexual and reproductive health advice.

## Introduction

Access to contraception and the ability to decide when and with whom to have a child are human rights (1). Moreover, the use of contraception can have a positive impact on maternal mortality and morbidity through its direct health benefits in preventing unintended pregnancies, and the subsequent likelihood of resorting to unsafe abortions (2). Still, 222 million women globally have an unmet need for modern contraception, and the need is greatest among socially vulnerable groups, such as adolescents (15–19 years) (2, 3). Recent global intergovernmental negotiations have acknowledged young people's (15–24 years) reproductive rights and stipulated the right to comprehensive sexuality education, access to sexual and reproductive health services, and the protection and promotion of young people's right to make their own choices regarding fertility control and sexuality (4).

Adolescents account for an estimated 16 million births annually, representing 11% of all births globally (5). Childbearing among young women as compared with older women is linked to higher maternal mortality ratios and maternal morbidity rates, as well as increased risk of induced, often unsafe abortions (6–8). Moreover, adolescent pregnancy negatively affects the child in terms of low birth weight, preterm delivery, and severe neonatal morbidity (9). Pregnancies among young women also force girls to compromise education or employment, which results in low educational attainment, poverty, and increased vulnerability (10). There is an identified need for a pronounced focus on contraceptive use, unintended pregnancy, and unsafe abortion among young women (10, 11). Moreover, there is a need to better understand how to communicate sexual and reproductive health messages to young people in general (12).

Uganda has one of the highest adolescent pregnancy rates in sub-Saharan Africa (13). Unintended pregnancy, the underlying cause of unsafe abortions, is common and more than 40% of births in Uganda are unintended (13, 14). Ugandan adolescents are at a higher risk of unintended pregnancies than adult women, and survey results indicate that 24% of adolescent girls are mothers or pregnant with their first child (13). Abortion is restricted in Uganda and unsafe abortions are common. An estimated 26% of maternal mortality is attributed to unsafe abortions, considered to be higher than the regional average (15, 16). Post-abortion care (PAC) is the emergency care provided to women with incomplete abortions. PAC includes post-abortion contraceptive counselling and provision as one of its core components (17). Although abortion is identified as a window of opportunity to motivate women to initiate contraception, it requires considerable effort and expertise to manage it effectively, and providers must be trained in counselling skills (18). Immediate uptake of contraception is crucial, since ovulation can return within 10 days of an abortion (19). Most contraceptive methods can be initiated immediately after an abortion, regardless of whether the abortion is medical or surgical (17). In spite of the introduction of contraceptive methods 50 years ago, the contraceptive prevalence in Uganda is low at 30%. Contraceptive use among young people is even lower; only 7% of adolescents (15–19 years) and 22% of young women (20–24 years) use modern contraception (13). This is in spite of the young age of sexual onset and the high prevalence of unintended pregnancies among young women (13). Healthcare providers (HCPs) play a major role in young people's access to contraceptive methods, and a study showed that Ugandan HCPs are reluctant to advise young people to use contraception and would not provide contraceptives to girls who are younger than 18 years; unmarried; still in school; or girls with no children (14).

There are Ugandan policies addressing adolescent health, including the National Adolescent Health Policy published in 2004 and updated in 2011, with the objective to mainstream adolescent health into the national development process (20). The policy aims to increase contraceptive use, encourage safe sex practices and abstinence, and improve access to adolescent-friendly services at health centres (20).

HCPs are essential in counselling and provision of quality reproductive health services to young people, which is why it is important to consider their attitudes towards sexually active young people (21). Previous studies map out HCP behaviour in surveys; however, little is known about HCP perceptions of their own role in contraceptive counselling to young people. The aim of this study is to explore HCP perceptions and practice regarding contraceptive counselling to young people.

## Methods

### Study design

This study used an inductive qualitative approach (22) and employed in-depth interviews (IDIs) to collect data. The nature of the interviews were open-ended, semi-structured questions following a topic guide that covered topics such as family planning, including PAC and contraceptive counselling and provision. The primary aim of the study was to explore HCP attitudes towards PAC; however, due to the open-ended questions and the emergent design of the study, the interviews centred on the responses of the participants and only used the topic guide as a point of departure. The results covering HCPs attitudes and perceptions of PAC have been published elsewhere (23). Due to the richness of the data and the fact that contraception among young people appeared to be an important topic for the interviewees, this paper presents the data covering HCP perceptions of contraceptive counselling use among young people.

### Study setting

The interviews were conducted in seven different health facilities that were located in rural, semi-urban, and urban settings, representing different levels of the healthcare system in Uganda (Table 1). The Ugandan healthcare system has several levels: the local level (village health teams, also called healthcare centre I), the district level (healthcare centre II, III, or IV, or district hospital), the regional level (regional referral hospital), and the national level (national referral hospital) (24). The seven health facilities were chosen due to their reported high caseload of PAC and hence the HCPs’ exposure to such cases.

**Table 1:** The location, setting, and level of each health facility, as well as the number and type of study participants interviewed from each facility

Facility	Setting/area	Level	Doctors interviewed	Midwives interviewed
Mpigi	Rural	Health centre IV	1	1
Luweero	Rural	Health centre IV	1	3
Nakaseke	Semi-urban	District hospital	1	4
Gombe	Semi-urban	District hospital	1	2
Entebbe	Urban	District hospital	2	2
Masaka	Urban	Regional hospital	2	3
Mulago	Urban	National hospital	2	2
Total			10	17

### Study participants

To be able to explore and better understand the working situation among HCPs at the district level in Uganda, we interviewed 10 doctors (including one specialist ob-gyn) and 17 midwives who were involved in PAC provision and contraceptive counselling or emergency obstetric care at their respective facilities. Standard PAC includes emergency care, contraceptive counselling, and treatment of and counselling for sexually transmitted infections (STIs) (17). However, current national standards and guidelines are rarely available at the facility level in Uganda and give little detail of what is included in the contraceptive counselling (25). The participants were recruited through the facility administration, who selected those who were exposed to PAC. However, they were also involved in other services at their respective facilities and there was no guarantee that the appointed participants actually did work with PAC or had any formal training in it. This is explained in detail in the published paper focusing on PAC (23).

### Data collection

Together with a Ugandan research assistant, the first author carried out the 27 IDIs with HCPs. Data were collected between February and March 2012 at seven health facilities situated in five different districts in the Central Region of Uganda. The interviews were conducted at the participants’ respective workplaces to maintain privacy as well as convenience. The topic guide was designed to explore the perceptions, skills, and competences of HCPs in relation to PAC and contraceptive counselling. The study design aimed to explore HCPs’ perceptions of PAC among all ages, including contraceptive counselling, social values, and attitudes towards reproduction. Within the latter topics, the situation of young people was specifically probed for and the discussion of young people permeated all topics discussed in the IDIs.

### Data analysis

For the analysis, the recorded data were transcribed verbatim and read through several times to identify codes and sub-themes related to contraception and young people, with a focus on young women. The data were structured using thematic analysis (26). The first and second authors manually generated codes and grouped them into categories using the scissors and paste technique (27). Discussions among all authors resulted in the identification of patterns, resulting in the emergence of two sub-themes and subsequently one main theme. This technique allows for the data to remain in context, while identifying semantic themes. This allows the findings to reflect broader meanings and implications of the data. Extracts were selected to illustrate the findings.

### Ethical considerations and approval

The topic is sensitive and carries relative stigma in Uganda; however, since the topics discussed mainly covered healthcare services and the role of a professional HCP, there were fewer ethical considerations. Nevertheless, it was important to create a safe and confidential environment for the participants to ensure their comfort. All participants were informed about the aim of the study, that their answers would be kept confidential, and that at any time they could withdraw from the study. All participants gave their written informed consent, where they also agreed that the interview could be audio recorded. Before conducting the interviews, an approval was obtained from each facility administration and head of department if applicable to ensure the cooperation of the facility beforehand. Ethical approval was obtained from the Makerere University College of Health Science, School of Biomedical Sciences Research and Ethics Committee, followed by approval from the Uganda National Council for Science and Technology.

## Results

**Fig. 1:**
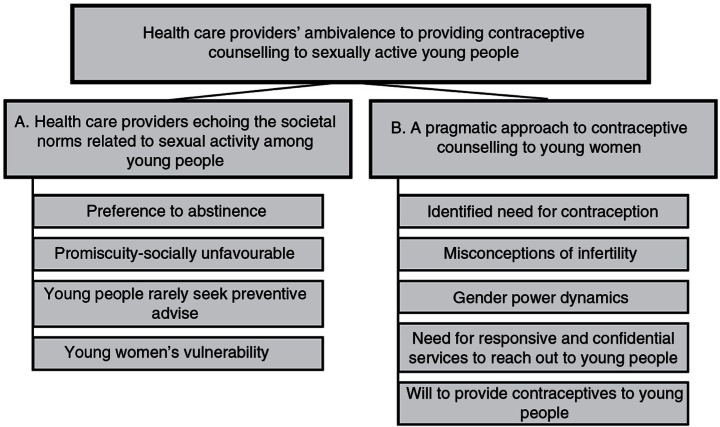
Thematic map illustrating the main theme, the sub-themes, and selected codes that led up to the sub-themes.

The main theme, HCPs’ ambivalence to providing contraceptive counselling to sexually active young people, emerged from the two sub-themes identified in analysis and illustrated in Fig. 1:

In the following section, the results from the two sub-themes are presented together with extracts from the data to illustrate the findings.

### A. HCPs echoing the societal norms related to sexual activity among young people

In the beginning of the interviews, the participants’ responses reflected general social norms when they talked about sexually active young people. The HCPs declared that young people should not engage in sexual activity. Sex was described as a biological act of reproduction and the participants meant that reproduction should only occur within the context of marriage. Therefore, young people should abstain from sex until they get married. Without probing for girls in particular during the interviews, the participants chose to focus on the appropriate behaviour of girls rather than that of boys. This focus is exemplified by the participants’ echoing the societal norms: respectable young women should avoid premarital sex.

I think that those young girls, they just need counselling. Just to abstain. I think abstinence is better for those young girls. (Midwife 19)

HCPs explained that men do not want to marry women who have already given birth. Further, they noted that a pregnant girl is usually expelled from school and would rarely return to school after giving birth. The preference for young people to practice abstinence resulted in society's, as well as to some extent the HCPs’, normative disapproval of contraceptive use, especially for unmarried young girls.

In spite of the initial condemnation of premarital sex, participants acknowledged that society is changing and that young people are engaging in sexual relationships, long-term or casual. However, the participants agreed that few of the sexually active young people would seek advice from the healthcare system due to the existing social stigma.

The young girls? Huh, they are not all that free to come because they fear their parents, they fear … they don't have time to take the pills, they even fear their relatives and friends seeing them taking the pills (…) But they don't come, many of them, because of the fear, but they would be many [if they didn't fear] (…). (Midwife 21)

Several participants mentioned the increase of transactional relationships, where young people trade sex for money to pay school fees or buy things. HCPs described girls as particularly vulnerable in such relationships due to their limited opportunities to negotiate condom use. Although some of the young girls sought advice from the health system to prevent pregnancy, the HCPs found the majority of unmarried girls among the women seeking PAC after an unsafely induced abortion rather than seeking contraceptive advice.

No, even those without children, like those schoolgirls, because even we have seen them coming for injection [DMPA], and they say, ‘I have my boyfriend, he is paying my school fees and I don't want to get pregnant as soon’. So they decide to come for family planning to prevent themselves from pregnancy, early pregnancies, hmm. (Midwife 20)

Although HCPs initially endorsed the existing socio-cultural values of upholding chastity, participants subsequently articulated concerns and understanding of the vulnerability of girls and the consequences of being pregnant at a young age, particularly when unmarried. The participants explained that the social stigma attached to pregnancies and use of contraception among young girls gave them no other choice than that of unsafe abortion in the event of an unintended pregnancy. Barriers such as embarrassment or fear of getting caught using contraception were thought to discourage young girls from seeking reproductive health services. Another barrier to contraceptive use among young girls was the social stigma, such as rumours of prostitution, attached to contraception use when unmarried.

But the relatives don't understand it that way. They feel if a child is using pills, she is a prostitute. She is having sex with many boyfriends, yet this is not the fact. Some [girls] they just want to have it [oral pill]. (Midwife 21)

### B. A pragmatic approach to providing contraceptive counselling to young women

As described in the previous sub-theme, the participants foremost encouraged abstinence in relation to contraception for young people. In terms of the participants’ role in contraceptive counselling, individual participants described their role as teaching young people to resist and control their sexual urges. However, the respondents also acknowledged that abstinence was not always possible. Hence, if a young person could not practice abstinence, HCPs should advice on condom use to avoid pregnancy and STIs. The majority of participants stated that condoms were the most appropriate contraceptive method for young people, partly due to their dual function of prevention of STIs and pregnancy. However, the participants recognised the issue of gender power imbalance and noted that young women may not have the power to negotiate condom use, especially in transactional relationships. Regardless of this, condoms were the preferred method due to their spontaneous, short-acting nature signalling that there is no continuous sexual activity planned, in contrast to long-acting reversible contraceptives (LARCs) such as intrauterine devices (IUDs) or the 3-month injectable (DMPA). Another prevalent concern among the participants regarding contraceptive use among young people was the uncertainty regarding appropriate methods for young people, other than condoms. Providers were unsure whether methods such as oral contraceptives, DMPA, and IUDs were suitable and safe for young women to use.

So, we say it's unfortunate that the family planning methods are not really styled up [suitable] for the young below like 15 [years]. You know that adolescent year. That time when you're 13–16, hmm. If there was a tendency where they'd say they have designed a family planning method for that age, you know it's healthy to give it to them [the young girls]. So that instead of doing an abortion, they are protected against getting pregnant. (Midwife 6)

Some participants, however, thought it would be better to give young people the possibility to choose from the whole spectrum of contraceptive methods, not only condoms, and to give them the correct knowledge to practice safer sex. They spoke of giving young people the opportunity to select the contraceptive method better suited for their needs.

So if the young people who also have a high sexual urge could know about contraception and then use it, since you can't stop them from having sex, then you can help them. So that they can have this sex in a safer way and that it does not result in effects that they don't want. Because they will go for sex, but they don't want the baby; they will go for sex but they don't want HIV. You see, those kinds of things, so it's [the lack of contraceptive awareness] a dilemma. (Doctor [senior Medical officer] 23)

Misconceptions regarding the possibility that contraceptive methods such as hormonal contraception or IUDs could result in infertility or cancer were prevalent among both midwives and doctors. These misconceptions persisted regardless of the woman's age; however, the HCPs were especially cautious in relation to young people or nulliparous women, since their fertility would be at stake. Therefore, providers advised women to have conceived at least once before initiating a contraceptive method in the case of infertility as a consequence of contraceptive use.

… For instance some family planning methods like your depo [DMPA] was meant for older women, 35 years plus, who have had a number of children. Because one of its problems is delayed return of fertility (…) I have seen many who have completely failed to conceive. So, earlier (…) it was not to be given to anyone without known fertility. (Doctor [ob-gyn] 15)

In addition, participants were afraid to be blamed for causing infertility due to the contraceptive advice given. This fear made HCPs reluctant to advise young nulliparous women to use a modern contraceptive method. Even participants who were sceptical about whether contraceptive use could actually result in infertility were too scared to advise young women to use it, in case the misconceptions were true. Doctors expressed concerns about the lack of knowledge among midwives in terms of contraceptive methods and explained that misconceptions about contraceptive use were common among midwives. However, doctors too articulated similar misconceptions misconceptions, indicating that they too would provide inappropriate methods or encourage abstinence that they would provide inappropriate methods or encourage abstinence instead of providing contraception.

[The midwife says] I can't [provide this contraception], but she doesn't have any reason. When you probe you find that she is saying maybe it causes cancer, which is not true but the perceptions [are wrong]. (Doctor [ob-gyn] 14)

Participants, especially the doctors, considered themselves to lack the appropriate counselling skills to effectively communicate with young clients and to provide accurate contraceptive advice. The participants found it difficult to address sex among young people in an appropriate way, and several felt uncomfortable discussing sex with young people. Individual HCPs mentioned the importance of a youth-friendly, non-judgmental, and sensitive approach when providing services to young women. Moreover, some providers highlighted the importance of confidentiality in service provision to young women. In the facilities included in the study, contraceptive counselling was primarily provided in the family planning unit in the family planning unit, often organised as a part of the antenatal care unit, where pregnant women come for check-up and examination. Hence, HCPs thought that unmarried women would not want to visit this unit to seek contraceptive counselling, however would have limited options to access such services. Moreover, it was the participants’ experience that the nurses working in antenatal care would not always be interested or motivated to counsel young girls. Instead, the antenatal care nurses would tell them off and send them home with neither advice nor contraception.

## Discussion

The main theme, *HCPs’ ambivalence to providing contraceptive counselling to sexually active young people*, reflects the conflict of personal and professional values HCPs experience in their encounter with young people. Moreover, the HCPs felt that they had inadequate knowledge to provide appropriate services in response to the young people's changing sexual behaviour. This study illustrates two aspects of young peoples’ access to sexual and reproductive health care: the health service provision *per se* and the existing social norms influencing HCPs’ decisions to act in a particular way during consultations with young people. The ambiguity among HCPs during consultations with young people with sexual and reproductive health needs in our study has also been observed among HCPs in relation to abortion care. Namely, their personal values conflict with service provision and providers are unprepared to provide appropriate services due to their lack of necessary skills and tools to deal with this dilemma (28). Moreover, we illustrate HCPs’ acknowledgement of young people's changing sexual behaviour and that in spite of their disapproving personal values, the HCPs in our study were willing to provide contraceptive services to unmarried and young people, under certain circumstances. Hence, young people's need for sexual and reproductive health services are being acknowledged and accepted to a certain extent from a professional health care perspective, yet disapproved of from a value-based personal perspective, creating ambiguities and frustrations among HCPs in their consultations with young people.

Our study, in accordance with previous studies, confirms the lack of implementation of the Adolescent Health Policy in terms of creating awareness among service providers concerning adolescent health at the facility level (14, 23, 29, 30). This lack may be interpreted as unwillingness within the health system to implement services that imply a social approval of sexual activity among young people. Our findings illuminate the existing mismatch between social norms as perceived by HCPs and existing policies to be enacted by HCPs. HCPs are seemingly left to provide services according to their best knowledge and capacity and not necessarily in line with evidence-based clinical guidelines. The apparent lack of guidelines, skills, and in-service management found in our study elucidates the faulty implementation of policies and makes contraceptive counselling and provision to young people particularly problematic. A recent study from Senegal calls the barriers based on age and marital status of clients seeking contraceptive counselling ‘provider-implemented’ due to lack of knowledge among providers (31). However, we argue that rather than being provider-implemented these barriers are an effect of the lack of implementation of existing policies, availability of guidelines in the clinics, and in-service management of reproductive health services, especially for young people. These notions are transferred to the young people, an example being that young sexually active Ugandan women report not being married as a major reason for not using contraception (13).

In addition to the inadequate implementation of existing policies, our study sheds light on the lack of organisation within the health facility in terms of sexual and reproductive health services. There was little organisational structure within the facilities to ensure contraceptive counselling to young people, especially in PAC. Health service barriers have been reported by young Ugandans as one of the main reasons for non-use of contraception (30). Thus, not having a routine of how to counsel young people who seek reproductive health services, or an assigned HCP to be in charge of such services, may not only complicate the provision of quality services, but is also likely to alienate young people from seeking advice at the health facilities.

HCPs reported infertility, cancer, or difficulties conceiving as potential side effects of contraceptive use, especially harmful for young people. These misconceptions transferred into fear of being held responsible for causing infertility – thus, the preference among our participants to advise nulliparous women to abstain. Previous research confirms that Ugandan HCPs believe that use of contraceptives early in life could have long-term side effects such as infertility (14). Moreover, the same study suggests that 45% of the HCPs lack adequate knowledge about contraceptives and that the misconceptions and safety concerns regarding certain contraceptive methods result in negative attitudes towards contraceptive counselling and provision to young people (14).

The results from our study indicate the need for clear evidence-based guidelines for HCPs and in-service training, including value clarification and attitude transformation, as well as improved in-service management to succeed with contraceptive counselling and provision to young people. Rehnström et al. argue that a more comprehensive pre-graduation curricula with regard to abortion care is needed (28). In line with this, we argue that contraceptive services with a specific focus on young people must be included in pre-graduation of HCPs. Such a measure could create an environment that is more tolerant of young people's sexuality and that recognises the beneficial public health effects of improved access to adolescent- and youth-friendly sexual and reproductive health services (32). It is crucial to consider both the health system and social aspects of sexual and reproductive health care when implementing policies on adolescent health and sexual and reproductive health care. Although this manuscript presents the views of HCPs regarding contraceptive counselling, it is worthwhile to mention young men's role in contraceptive use in this context. Interestingly, the HCPs did not specifically discuss young men, but referred to young people in general. All experiences the participants referred to were based on consultations with young girls, underlining the absence of men involved in contraceptive counselling and reproductive healthcare seeking. A recent study suggests that counselling men on contraceptive methods may empower them to become more actively involved in contraceptive decisions (33). This finding underlines the importance of increasing the efforts to reach out to young men in the Ugandan context to increase their awareness and empower them to take an active role in the contraceptive dialogue.

Our study shows various levels of contraceptive services that need improvement. Importantly, we show HCPs’ understanding of the importance of such services and their willingness to improve. Now the Ugandan health system and policymakers need to decide whether the required interventions to implement existing policies and improve contraceptive services are to be enacted or not.

### Methodological considerations

This study was conducted with the aim of elucidating HCPs’ perceptions of PAC and hence included providers with experience in PAC. The participants’ exposure to post-abortion cases may have added to their awareness of young women's situations and need for sexual and reproductive health services and hence may have influenced the perceptions of the participants. However, given that the answers initially reflected what is socially accepted in Uganda and became more elaborative and open towards the end of the interview, we believe that the data do reflect the situation in health facilities in Uganda today and probably also in similar settings. Moreover, since the study was carried out at different levels of the health system and in different settings, we believe that our findings reflect perceptions among HCPs of the situation of young women in Uganda with regard to contraception and sexual and reproductive healthcare needs, enhancing the transferability of this study. Given that the main interviewer was a young Swedish woman and the interviews were conducted in a setting where privacy was ensured, the HCPs felt comfortable talking about controversial topics, judging from the nature of the data obtained. The Ugandan research assistant was helpful in interpreting expressions and broaching difficult topics and added input to the content of the interviews and their interpretation.

## Conclusions

Health care providers are not sufficiently equipped to provide contraceptive counselling to the young girls and women that they encounter. Social norms and personal values contradict professional tasks. The lack of necessary skills and support from the health system impedes provision of adequate and correct contraceptive counselling to young people, especially to young women, since the men are generally absent from the health facilities. In order to improve this situation, a clear policy followed by a clear strategy with practical guidelines on contraceptive counselling is needed. Moreover, this must be implemented along with in- and pre-service training including value clarification, stigma reduction, and attitude transformation to equip providers to better cater to young people. Additionally, to improve access to contraception among young people, improved facility management of reproductive health services targeting young people (including men) is crucial. Together with available structures for supervision, these measures could lead to motivated health workers able to provide a stigma-free environment and promote sexual and reproductive health information and services to young people.
